# Effectiveness of molnupiravir for treating COVID-19 in patients with psychiatric disorders

**DOI:** 10.3389/fphar.2024.1384264

**Published:** 2024-07-04

**Authors:** Ting-Hui Liu, Hsuan-Yi Liao, Chih-Cheng Chang, Chih-Cheng Lai

**Affiliations:** ^1^ Department of Psychiatry, Chi Mei Medical Center, Tainan, Taiwan; ^2^ School of Medicine, College of Medicine, National Sun Yat-sen University, Kaohsiung, Taiwan; ^3^ Department of Intensive Care Medicine, Chi Mei Medical Center, Tainan, Taiwan; ^4^ School of Medicine, College of Medicine, National Sun Yat-sen University, Kaohsiung, Taiwan

**Keywords:** COVID-19, hospitalization, mortality, molnupiravir, psychiatric disorder, SARS-CoV-2, post-COVID-19 condition

## Abstract

**Objectives:**

This study investigated the clinical effectiveness of molnupiravir for treating non-hospitalized COVID-19 patients with pre-existing psychiatric disorder.

**Methods:**

This retrospective cohort study used the TriNetX research network to identify patients with psychiatric disorder who experienced non-hospitalized COVID-19 between 1 January 2022, and 1 May 2023. The propensity score matching (PSM) method was used to match patients receiving molnupiravir (treated group) with those who did not (untreated group). The outcome included short-term outcomes - the composite of all-cause hospitalization or death within 30 days and the risk of post-COVID-19 conditions up to a year after COVID-19 diagnosis.

**Results:**

Two groups of 9,421 patients, each with balanced baseline characteristics, were identified using the PSM method. During the 30-day follow-up, treated group was associated with a reduced risk of hospitalization or mortality compared to untreated group (HR, 0.760; 95% CI, 0.665–0.869). Compared to untreated group, treated group also exhibited a decreased risk of experiencing post-COVID-19 conditions, including chest/throat pain (HR, 0.615; 95% CI, 0.543–0.696), abnormal breathing (HR, 0.761; 95% CI, 0.687–0.884), abdominal symptoms (HR, 0.748; 95% CI, 0.674–0.831), fatigue (HR, 0.718; 95% CI, 0.638–0.808), headache (HR, 0.753; 95% CI, 0.665–0.852), cognitive symptoms (HR, 0.769; 95% CI, 0.630–0.940), myalgia (HR, 0.647; 95% CI, 0.530–0.789), cough (HR, 0.867; 95% CI, 0.770–0.978), and palpitation (HR, 0.641; 95% CI, 0.534–0.770) during the 1-year follow-up.

**Conclusion:**

Molnupiravir could be associated with lower rates of all-cause hospitalization or death and also lower risk of post-COVID-19 condition among non-hospitalized COVID-19 patients with pre-existing psychiatric disorder.

## 1 Introduction

COVID-19, caused by SARS-CoV-2, emerged as a global pandemic in late 2019 (Lai et al., 2020). This highly transmissible respiratory pathogen has significantly impacted global health systems. As of 22 November 2023, over 772 million confirmed cases, resulting in 6,981,263 deaths, have been reported to the World Health Organization. While most SARS-CoV-2 infections present as mild-to-moderate diseases, older individuals and those with underlying conditions such as immunocompromised states, cancer, sickle cell disease, chronic kidney disease, chronic liver disease, cardiovascular diseases, organ transplant recipients, autoimmune or inflammatory conditions, HIV infection, and other conditions affecting the immune system or blood may face a higher risk of COVID-19 progression.

Notably, psychiatric disorders emerge as another significant risk factor for severe COVID-19 (Cheng et al., 2023). A retrospective cohort study involving 15,783 adult patients found that, compared to non-psychiatric patients, those with psychiatric disorders had higher rates of severe COVID-19, hospitalization, and a shorter duration to in-hospital mortality (Cheng et al., 2023). Additionally, the study by Taquet et al. highlighted that individuals with psychiatric disorders were more likely to experience severe COVID-19 symptoms and prolonged recovery periods (Taquet et al., 2021b). This elevated risk is further supported by Goldberger et al., who reported that psychiatric patients had nearly twice the mortality rate of non-psychiatric patients when infected with COVID-19 (Goldberger et al., 2022). Another study by Koyama et al. demonstrated that psychiatric disorders, particularly depression and anxiety, were associated with a higher likelihood of ICU admission and mechanical ventilation among COVID-19 patients (Koyama et al., 2022). These findings showed the importance of antiviral agents in preventing severe COVID-19 outcomes for patients with existing psychiatric diseases.

Currently, two oral antiviral agents, molnupiravir and nirmatrelvir plus ritonavir, have been developed for high-risk individuals with mild-to-moderate COVID-19. Their efficacy in preventing severe SARS-CoV-2 infections has been demonstrated in randomized controlled trials (Hammond et al., 2022; Jayk Bernal et al., 2022)and numerous real-world studies (Wong et al., 2022; Evans et al., 2023; Najjar-Debbiny et al., 2023a; b; Wai et al., 2023; Yip et al., 2023). However, studies specifically assessing the effectiveness of these two antiviral agents in treating COVID-19 in patients with psychiatric disorders are scarce. While a previous network meta-analysis suggested that oral nirmatrelvir plus ritonavir might be more effective than molnupiravir in preventing severe COVID-19 (Lai et al., 2022), concerns arise in clinical practice due to the intricate interaction between nirmatrelvir plus ritonavir and psychotropic medications (Chatterjee et al., 2023). In contrast, molnupiravir poses a lower risk of drug-drug interactions with psychotropic medications, making it a relatively safe anti-SARS-CoV-2 agent for patients with psychiatric diseases. Therefore, this study was conducted to evaluate the therapeutic efficacy of molnupiravir in individuals with pre-existing psychiatric diseases and COVID-19.

## 2 Materials and methods

### 2.1 Data source

This retrospective cohort study used data from the TriNetX research network, which is a global health-collaborative clinical-research platform collecting real-time electronic medical data from a network involving more than 250 million patients from more than 120 healthcare organizations (HCOs) across 19 countries in North and South America, Asia-Pacific, Europe, the Middle East, and Africa (TriNetX, 2023). These records encompass a wide variety of patient information, including demographic details, medical diagnoses, procedures, medication records, laboratory results, genomic data, and types of healthcare organization (HCOs) visits. TriNetX offers integrated tools for patient-level data analysis and delivers aggregated results to the researchers (TriNetX, 2023). Detailed information on the database can be accessed online. Written informed consent was not required because TriNetX contains anonymized data. The Institutional Review Board of the Chi Mei Medical Center approved the study protocol (no. 11202–002).

### 2.2 Patient selection

First, we selected a cohort of adult patients with pre-existing psychiatric disorders who also had COVID-19 from 1 January 2022 to 1 May 2023. These psychiatric disorders were defined according to the International Classification of Diseases, Tenth Revision, Clinical Modification (ICD-10-CM) code. These codes include psychotic (F20–F29), mood (F30–F39), and anxiety (F40–F48) disorders. Specifically, psychotic disorders fall under the classification codes F20 through F29. F20 refers to Schizophrenia, F21 is for Schizotypal disorder, F22 signifies Persistent delusional disorders, F23 denotes Acute and transient psychotic disorders, F24 is the code for Induced delusional disorder, F25 represents Schizoaffective disorders, F28 is for Other nonorganic psychotic disorders, and lastly, F29 is used for Unspecified nonorganic psychosis. The diagnosis of COVID-19 or a recorded positive PCR test for COVID-19 on the basis of the ICD-10-CM code U07.1 (“COVID-19”) or a positive SARS-CoV-2 and related RNA laboratory test result (TNX: LAB:9088), as previously described (Hsu et al., 2023c; Liu et al., 2023c). To make sure the patients had regular follow-up, only patients having at least two medical visits with HCOs from 1 January 2022, to 1 May 2023. In contrast, we excluded patients receiving other recommended treatments for non-hospitalized patients with COVID-19, including remdesivir, nirmatrelvir plus ritonavir, hospitalized patients with COVID-19 within 5 days on or after SARS-CoV-2 infection, and patients with post-COVID-19 related symptoms before index date. Afterall, we divided the included patients into two cohorts: those receiving molnupiravir (the treated group) and those not receiving molnupiravir (the untreated group). The demographic characteristics (i.e., age, sex, race, and socioeconomic status), comorbidities (hypertensive diseases, neoplasms, chronic lower respiratory diseases, overweight/obesity, type 2 diabetes mellitus, chronic kidney diseases, chronic liver disease, cardiovascular diseases and nicotine dependence) of the included patients were collected as previous studies (Hsu et al., 2023a; Tsai et al., 2023; Wu et al., 2023).

### 2.3 Outcome

In this study, we measured the short-term outcomes - the composite of all-cause hospitalization or death within 30 days and the risk of post-COVID-19 conditions up to a year after COVID-19 diagnosis. The post-COVID-19 conditions were defined as the presence of any of the following ten symptoms, including chest/throat pain, abdominal symptoms, fatigue, headache, cognitive symptoms, myalgia, sleep disturbance, cough, and palpitation, using ICD-10 codes, as previous studies (Taquet et al., 2021a; Liu et al., 2023a; Liu et al., 2023b).

### 2.4 Statistical analysis

The characteristics of the two groups are presented as mean ± standard deviation (SD), frequency, and proportion. To balance the distribution of covariates between the groups at baseline, PSM was performed using a nearest-neighbor greedy matching algorithm with a caliper width of 0.1 pooled SDs. A standardized difference of <0.1 between the two groups was indicative of appropriate matching on any variable (Haukoos and Lewis, 2015). After PSM, the incidence of each outcome was estimated using the Kaplan-Meier analyses combined with log-rank tests. The results were expressed as hazard ratios (HRs) with corresponding 95% confidence intervals (CIs). The threshold for statistical significance was set at *p* < 0.05. All statistical analyses were performed using built-in functions of the TriNetX platform.

## 3 Results

### 3.1 Demographic characteristics of included patients

**FIGURE 1:**
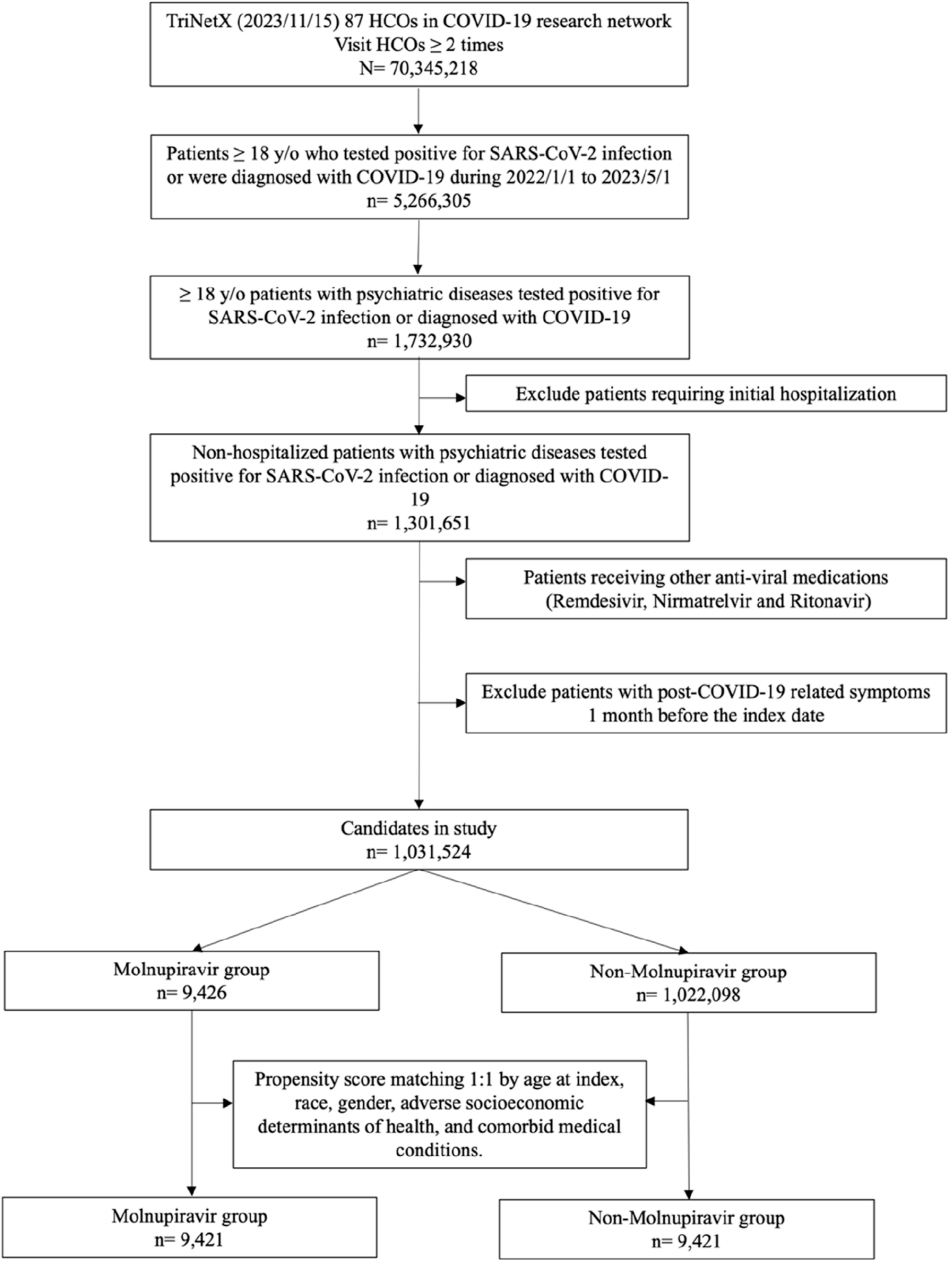
The algorithm of patient selection and cohort construction.

Initially, a total of 70, 345, 218 individuals with more than two times of HCO visits were identified from TriNetX on 15 November 2023. Among them, 1,301,651 non-hospitalized patients with COVID-19 and pre-existing psychiatric diseases were found (Figure 1). After excluding patients receiving remdesivir and nirmatrelvir plus ritonavir, and those with previous post-COVID-19 related symptoms, 1,031,524 patients were included, in which 9,426 and 1,022,098 patients were classified as the treated and untreated group, according to the use of molnupiravir (Figure 1). Compared with the untreated group, the treated group was older, had more white and fewer Black or African-American patients, and had a higher proportion of patients with hypertensive diseases, neoplasms, overweight/obesity, type 2 diabetes mellitus, chronic lower respiratory disease, chronic kidney disease, ischemic heart diseases, asthma, and fatty liver (all SDs >0.1). Through PSM, we identified two well-matched groups, each consisting of 9,421 patients, and all the differences in the demographic features were minimalized (all SDs <0.1) (Table 1).

**TABLE 1:** Characteristics of patients with and without Molnupiravir administration before and after propensity score matching.

	Before matching			After matching		
Characteristic name	Molnupiravir group (*n* = 9,426)	Non-molnupiravir group (*n* = 1,022,098)	Std diff	Molnupiravir group (*n* = 9,421)<	Non-molnupiravir group (*n* = 9,421)	Std diff
Age at Index	61.5±15.3	48.6±19.0	0.751	61.5±15.3	61.8±15.7	0.019
Female	5897 (62.56)	663364 (64.92)	0.049	5893 (62.55)	5832 (61.9)	0.013
Male	3317 (35.19)	326908 (31.99)	0.068	3316 (35.2)	3382 (35.9)	0.015
Race						
White	7957 (84.42)	715104 (69.98)	0.349	7952 (84.41)	7979 (84.69)	0.008
Black or African American	708 (7.51)	141740 (13.87)	0.207	708 (7.52)	700 (7.43)	0.003
Asian	158 (1.68)	20700 (2.03)	0.026	158 (1.68)	85 (0.9)	0.069
Other Race	62 (0.66)	40138 (3.93)	0.220	62 (0.66)	179 (1.9)	0.011
Problems related to housing and economic circumstances	991 (10.51)	22489 (2.2)	0.346	986 (10.47)	890 (9.45)	0.034
Comorbidities						
Hypertensive diseases	6090 (64.61)	358770 (35.11)	0.617	6085 (64.59)	6205 (65.86)	0.027
Neoplasms	3894 (41.31)	222789 (21.8)	0.429	3889 (41.28)	3860 (40.97)	0.006
Overweight and obesity	3178 (33.72)	216015 (21.14)	0.285	3175 (33.7)	3237 (34.36)	0.014
Type 2 diabetes mellitus	2934 (31.13)	158346 (15.5)	0.376	2930 (31.1)	2922 (31.02)	0.002
Chronic lower respiratory diseases	2897 (30.73)	209413 (20.49)	0.236	2895 (30.73)	2902 (30.8)	0.002
Chronic kidney disease	2438 (25.87)	84913 (8.31)	0.480	2433 (25.83)	2411 (25.59)	0.005
Ischemic heart diseases	2131 (22.61)	99482 (9.74)	0.355	2128 (22.59)	2084 (22.12)	0.011
Asthma	1774 (18.82)	137266 (13.43)	0.147	1773 (18.82)	1817 (19.29)	0.012
Nicotine dependence	1300 (13.79)	144464 (14.14)	0.010	1300 (13.8)	1578 (16.75)	0.082
Fatty liver	705 (7.48)	37617 (3.68)	0.166	704 (7.47)	703 (7.46)	0.000
Fibrosis and cirrhosis of liver	249 (2.64)	14140 (1.38)	0.090	248 (2.63)	224 (2.38)	0.016
Alcoholic liver disease	57 (0.61)	5348 (0.52)	0.011	57 (0.61)	46 (0.49)	0.016

### 3.2 Outcome of interests

**FIGURE 2:**
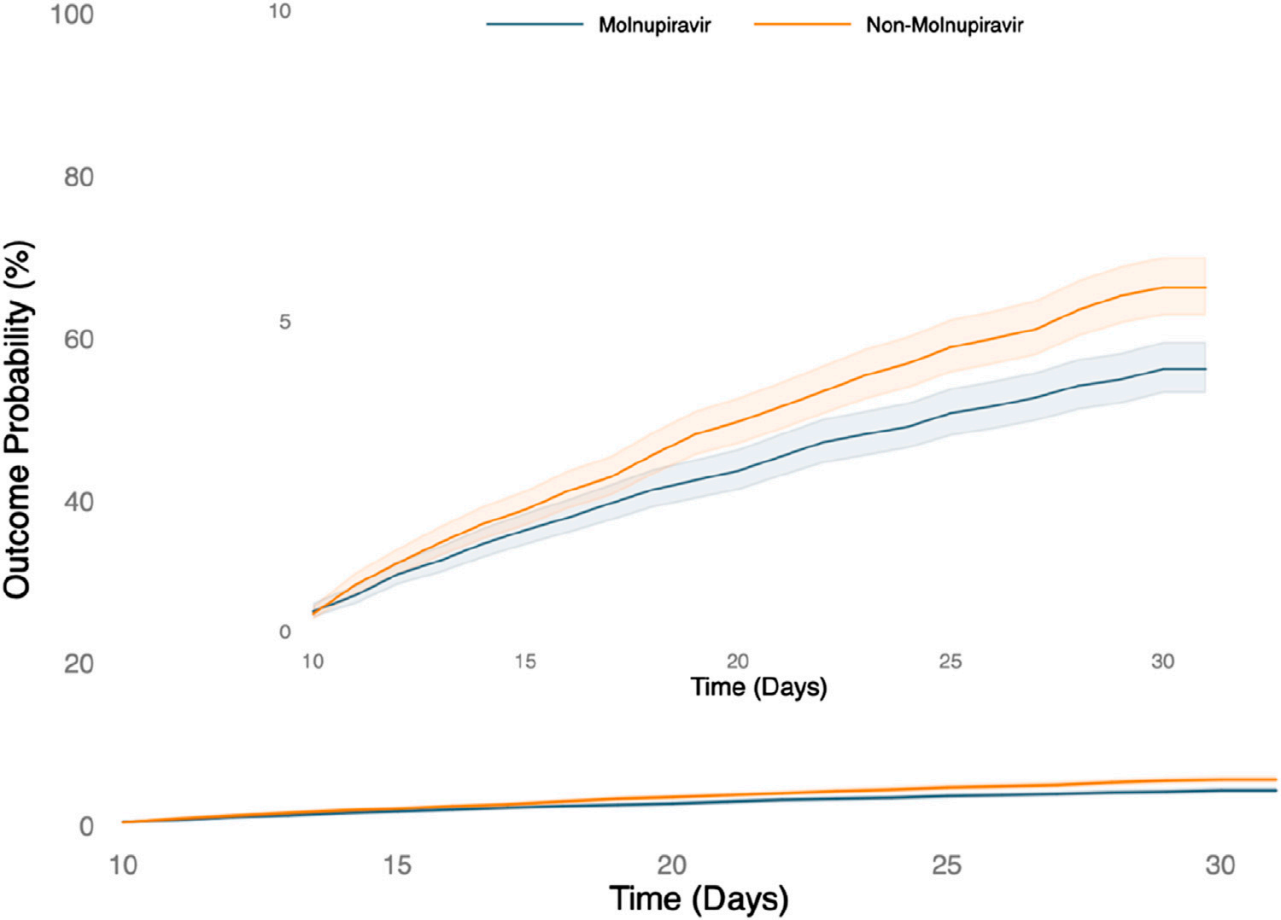
The probability of the short-term outcome-composite outcome of all-cause hospitalization or death within 30 days.

**FIGURE 3:**
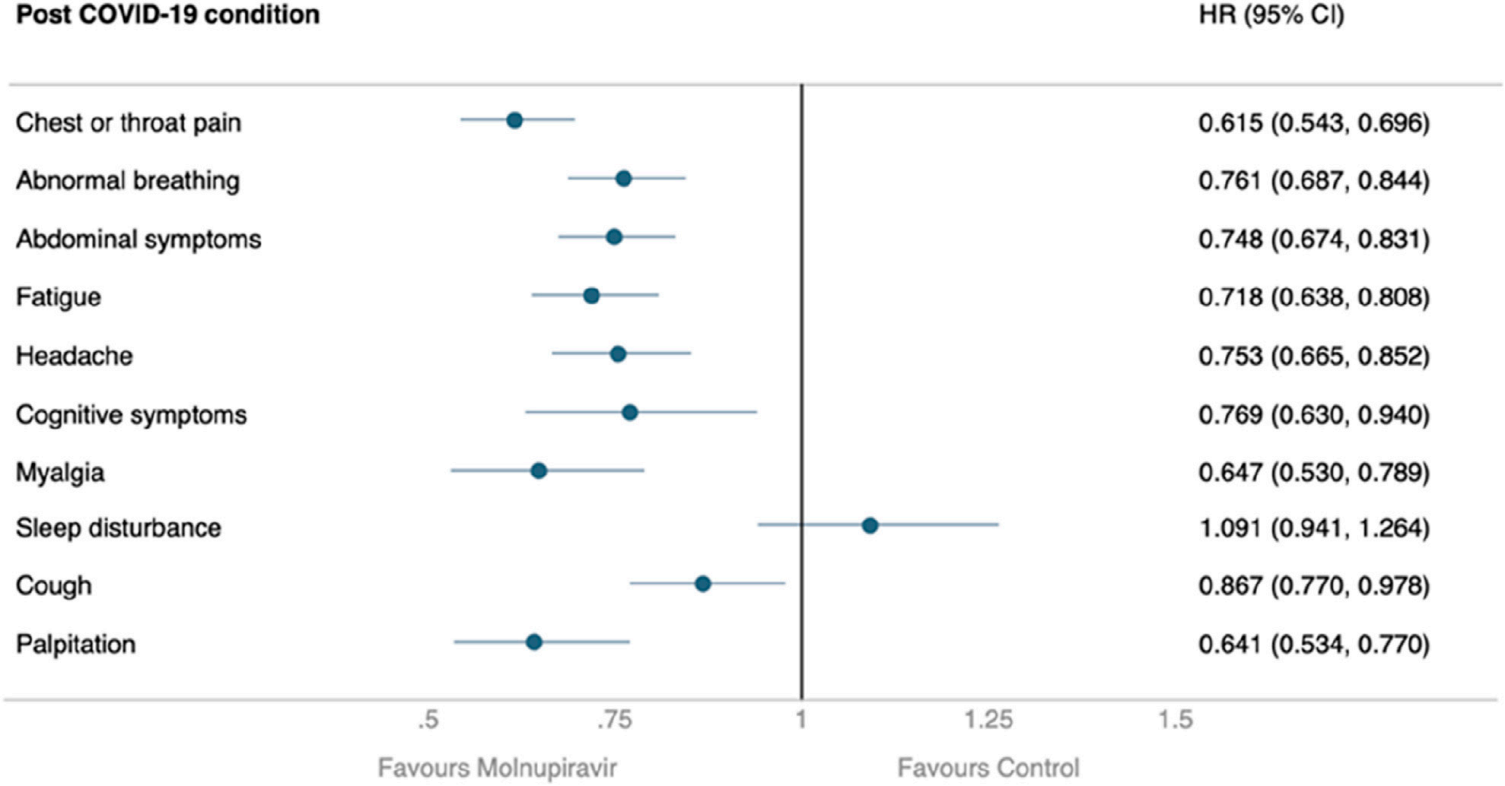
Long term outcome as Post COVID-19 condition within 90–180 days after index date.

During the 30-day follow-up, patients receiving molnupiravir were associated with a reduced risk of hospitalization or mortality compared to those who did not (HR, 0.760; 95% CI, 0.665–0.869). Similarly, the treated group had a lower probability of composite outcome of all-cause hospitalization or death within 30 days than the untreated group (log-rank test, *p* < 0.001) (Figure 2). Regarding post-COVID-19 condition, compared to those not receiving molnupiravir, patients treated with molnupiravir exhibited a decreased risk of experiencing post-COVID-19 conditions, including chest/throat pain (HR, 0.615; 95% CI, 0.543–0.696), abnormal breathing (HR, 0.761; 95% CI, 0.687–0.884), abdominal symptoms (HR, 0.748; 95% CI, 0.674–0.831), fatigue (HR, 0.718; 95% CI, 0.638–0.808), headache (HR, 0.753; 95% CI, 0.665–0.852), cognitive symptoms (HR, 0.769; 95% CI, 0.630–0.940), myalgia (HR, 0.647; 95% CI, 0.530–0.789), cough (HR, 0.867; 95% CI, 0.770–0.978), and palpitation (HR, 0.641; 95% CI, 0.534–0.770) during the 1-year follow-up (Figure 3).

## 4 Discussion

This first study assessed the effectiveness of molnupiravir for treating non-hospitalized COVID-19 patients with pre-existing psychiatric disorder. Our findings indicated that molnupiravir could be linked to a decreased risk of short-term hospitalization and mortality, as well as a reduced risk of subsequent post-COVID-19 conditions. Specifically, among patients with pre-existing psychiatric disorders, molnupiravir lowered the risk of all-cause hospitalization or mortality by 24% during the 30-day follow-up. Furthermore, it contributed to a decline in the development of post-COVID-19 symptoms, including chest/throat pain, abnormal breathing, abdominal symptoms, fatigue, headache, cognitive symptoms, myalgia, cough, and palpitation during the 1-year follow-up.

Our findings align with prior studies demonstrating the short- and long-term benefits of molnupiravir in treating patients with mild-to-moderate COVID-19 (Bajema et al., 2023; Hsu et al., 2023b). Bajema et al. highlighted the efficacy of molnupiravir in reducing viral load and improving recovery times in general populations, while Hsu et al. showed its role in decreasing hospitalization rates and mortality among COVID-19 patients. Additionally, a study by Xie et al. (2023) emphasized molnupiravir’s effectiveness in lowering the incidence of long COVID symptoms , which aligns with our findings of reduced post-COVID-19 conditions such as chest/throat pain, abnormal breathing, and fatigue. Notably, this study focuses on patients with psychiatric disorders, a demographic often underrepresented in clinical research, thereby expanding the treatment spectrum of molnupiravir. Previous research by Korzeniowska et al. (2023) indicated that individuals with psychiatric disorders face higher risks of severe COVID-19 outcomes, making our findings particularly relevant. By demonstrating the safety and efficacy of molnupiravir in this specific high-risk group, our study endorses its use and advocates for more inclusive treatment protocols. This is supported by De Rose et al. (2023), who also emphasized the need for tailored therapeutic strategies for patients with psychiatric conditions, recognizing their unique vulnerabilities during the pandemic. Consequently, our study not only corroborates existing evidence but also provides new insights into the management of COVID-19 among patients with psychiatric disorders, advocating for broader adoption of molnupiravir in clinical practice.

Our findings carry significant clinical implications. Given the elevated risk of COVID-19 progression in patients with psychiatric disorders (Cheng et al., 2023), the prompt and appropriate use of antiviral agents becomes crucial in preventing the development of severe COVID-19. While a previous study identified nirmatrelvir plus ritonavir as the primary drug choice for the patients with mild-to-moderate COVID-19 (Lai et al., 2022), and more effective than molnupiravir, concerns remain regarding its potential drug-drug interactions with antipsychotic medications in patients with psychiatric disorders (Chatterjee et al., 2023). Consequently, our findings offer robust evidence regarding the clinical effectiveness of molnupiravir in patients with psychiatric disorders, underscoring its significance within this specific high-risk group.

Although the present and previous studies (Wong et al., 2022; Evans et al., 2023; Najjar-Debbiny et al., 2023a; Najjar-Debbiny et al., 2023b; Wai et al., 2023; Yip et al., 2023) have demonstrated the clinical benefits of oral antiviral agents for treating COVID-19 in high-risk patients, our findings reveal that only a small proportion of patients with both COVID-19 and psychiatric disorders received molnupiravir. After excluding patients receiving nirmatrelvir plus ritonavir or remdesivir, only less than 1% (*n* = 9426) among the 1,031,524 patients with psychiatric disorders in this study received. This finding underscores the importance for healthcare authorities to invest additional efforts in improving compliance and appropriately prescribing oral antiviral agents for high-risk patients, especially those with psychiatric disorders.

This study had several strengths. Its real-world design, encompassing a large and diverse population, enhances the applicability of its findings to real-life scenarios. The use of PSM to align demographic features between the treated and untreated groups helps mitigate potential confounding effects. Finally, the study was conducted between January 2022 and May 2023, during the predominance of the Omicron variant. Consequently, our findings hold relevance to the current stage of the pandemic.

This study had certain limitations. The utilization of electronic health records introduces the possibility of coding errors, potentially leading to inaccuracies during data collection and analysis. This could impact the robustness of the study’s conclusions. Additionally, despite efforts to control various variables, residual confounding remains a challenge in real-world studies, acknowledging potential influences that were not entirely accounted for in the analysis. Lastly, we did not assess the effect of molnupiravir for each specific psychiatric disorder; however, further study is warranted to explore this aspect in detail.

## 5 Conclusion

In summary, our study indicates that molnupiravir can effectively reduce all-cause hospitalizations and deaths in COVID-19 patients with psychiatric conditions. Furthermore, it demonstrates significant benefits in long-term outcomes, including a decreased risk of post-COVID-19 conditions within 1 year after a COVID-19 diagnosis. These findings support the use of molnupiravir in managing COVID-19 in patients with psychiatric conditions.

## 6 Declarations

### 6.1 Ethical approval

Written informed consent was not required because TriNetX contains anonymized data. The Institutional Review Board of the Chi Mei Medical Center approved the study protocol (no. 11202–002).

## Data Availability

The original contributions presented in the study are included in the article/Supplementary material, further inquiries can be directed to the corresponding author.
